# Tsai Chiao: The founder of physiology and aviation, aerospace and navigation medicine in China

**DOI:** 10.1007/s13238-020-00737-3

**Published:** 2020-05-30

**Authors:** Yalan Wang, Zijian Li, Yanyan Qian, Benyu Guo

**Affiliations:** 1 Psychology College, Nanjing Normal University, Nanjing, 210097 China; 2 Social and Behavioural Sciences Faculty, Leiden University, 2333 AK Leiden, Netherlands

**Figure 1:**
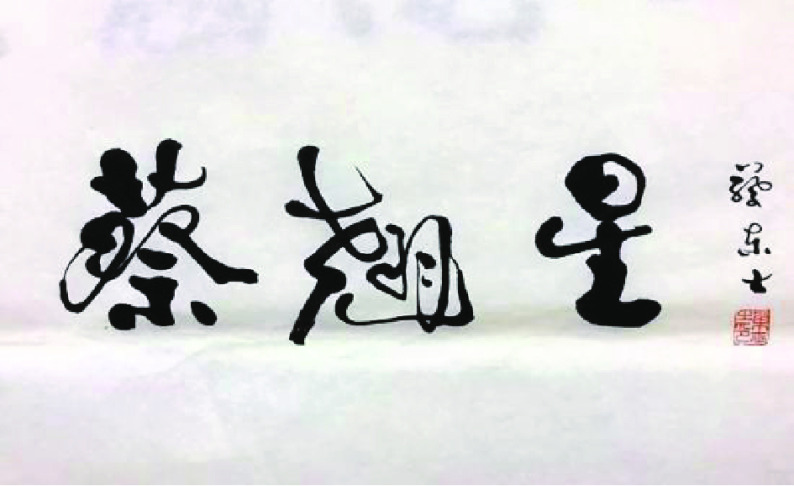
The name of minor planet Cai Qiao stem from Chinese physiologist Tsai Qiao

On February 16th, 2019, Tsai Academician Museum held a launch ceremony of a minor planet (207681)’s settling in Jieyang County, Guangdong Province. This minor planet was given a new name Cai Qiao (Fig. 1) on October 14th, 2011. Cai Qiao was discovered at Xu Yi Station in the Purple Hills Observatory of the Chinese Academy of Sciences on August 16th, 2007. So who is the planet named after?

**Figure 2:**
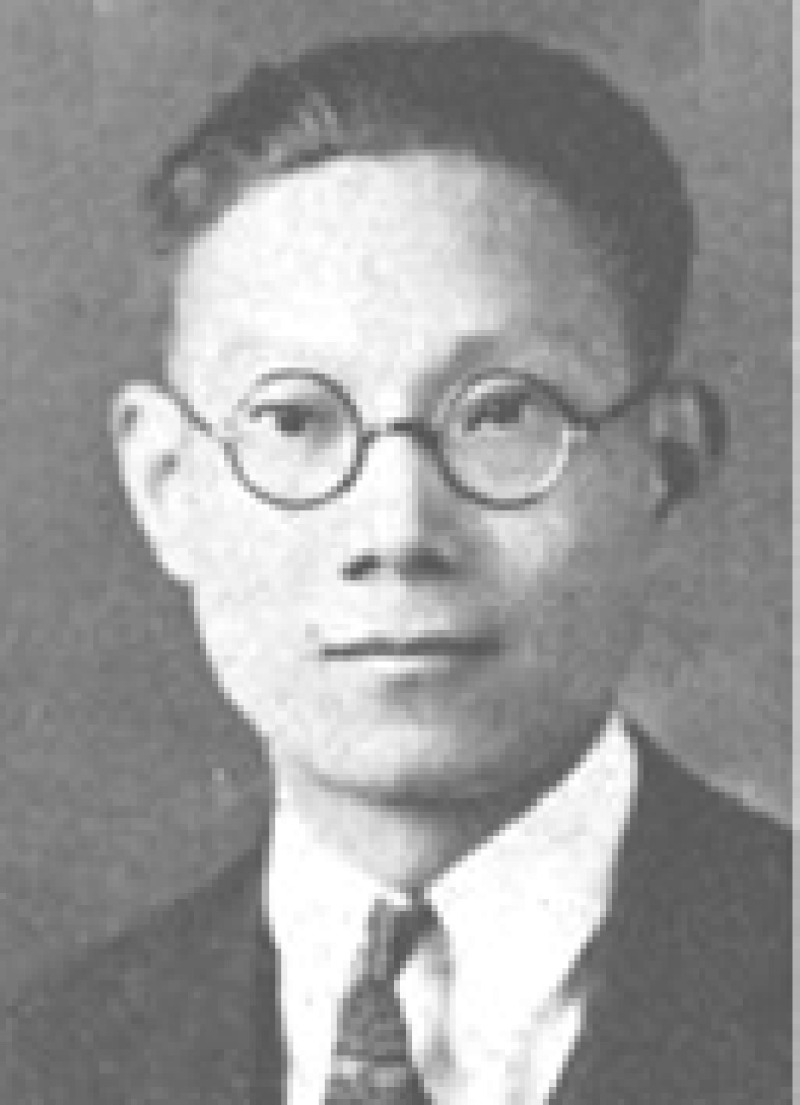
Tsai Chiao

Tsai Chiao (蔡翘, 1897–1990) (Fig. 2) styled name was Zhuofu (卓夫), infant name was Yizhong (义忠). As one of the founders of Chinese physiology, Tsai promoted the development of Chinese physiology and also furthered the prosperity of international neuroanatomy. He was also the originator of China's aviation, aerospace and navigation medicine, who initially established China’s aviation physiological system.

October 11th, 1897, Tsai was born in Xianmei Country, Xinheng Town, Jiedong Distinct, Jieyang City, Guangdong Province. Tsai went to an old-style private school when he was 7, and subsequently transferred to Lam Tin Government Elementary School. In 1913, he received education from Jinshan Academy located at Chaoan County, Guangdong Province, and graduated in 1917. In 1918, he received further education from Shanghai Fudan University Attached Middle School to improve his English. The same year, he became an external student of the Chinese Department of Peking University. In the autumn of 1919, deeply influenced by the cultural trend “Saving the Country through Science” of May 4th Movement, he went to America at his own expense.

**Figure 3:**
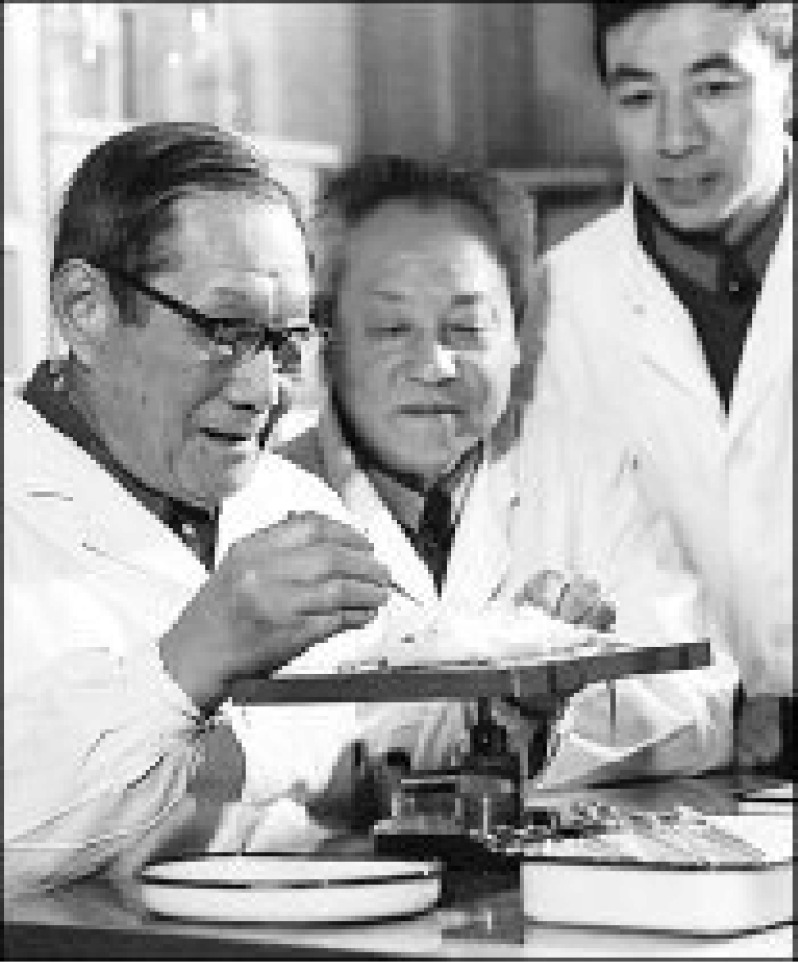
Tsai (first from the left) and the technicians were discovering the ventral mesencephalic tegmentum

Affected by behavioral psychologist Zing-Yang Kuo (郭任远) (Qian et al., 2018), Tsai studied a regular college course for two years, firstly at Psychology Department, California University, and then transferred to the same department at Indiana University. In the winter of 1921, he entered the University of Columbia as a postgraduate. From 1922 to 1925, he transferred to the Department of Physiology, College of Arts and Sciences, University of Chicago to take more postgraduate courses. During this period, Professor Harvey A. Carr of comparative psychology supervised Tsai’s study and Tsai majored mainly in psychology, minored in physiology and neuroanatomy. In 1924, Tsai gained a philosophic doctor degree with the doctoral thesis *A comparative study of retention curves for motor habits* in which he further investigated the differences of memory retention between white rats and humans on a stylus maze problem (Tsai, 1924). In 1925, at the Hull Laboratory of Anatomy of Chicago University (Fig. 3), Tsai explored “the optic tracts and centers of the opossum (Tsai, 1925a)” and described its “descending tracts and related structures of the thalamus and midbrain (Tsai, 1925b)”. In his exploration, Tsai described in great detail the ventral mesencephalic tegmentum where the nucleus pretectalis is located. This area is called Tsai’s area or ventral area of Tsai. In addition, Tsai proved the fact that “the normal food-seeking impulse in the albino rat as measured by the choice method is stronger than that of sex (Tsai, 1925c)” against the Freudian conception that the sex urge is the strongest of all human motives. The same year Tsai was awarded Chicago University’s Gold Key Award and recommended as a member of the American Association of Anatomists.

In the autumn of 1925, Tsai returned back to China. With the support of Zing-Yang Kuo, the vice-president of Fudan University, Tsai constructed biology discipline at Psychology College, Fudan University, taught biology and physiology, and cultivated a bunch of researchers like Yanfeng Hsü (徐丰彦), Depei Feng (冯德培), Henian Chu (朱鹤年). In 1927, Tsai served as a Physiology Professor in the Medical Colleague of National Central University in Shanghai. Tsai acted as editor of the first textbook called *Physiology*, the first textbook of physiology in China, which was published in 1929 as part of a college book series. After that, Tsai also compiled 10 more Chinese physiology books including *Physiology Experiment*, *Exercise Physiology* and the *General Knowledge of Physiology*. During this period, Tsai also published 8 papers concerning the issue of parathyroid. Firstly, co-operating with his teaching assistant Hsü, Tsai explored the relationship between parathyroid tetany and the removal of the large intestine (Tsai and Hsü, 1929a), ligation of the bile duct (Tsai and Hsü, 1929b), intestinal obstruction (Tsai and Hsü, 1929c) and intestine putrefaction (Tsai and Hsü, 1929d). Secondly, they studied plasma calcium and inorganic phosphorus following intravenous injections (Tsai and Hsü, 1930a; Tsai and Hsü, 1930b), and the calcium content of skeletal muscles after thyroparathyroidectomy and parathormone injections (Hsü and Tsai, 1930). Finally, he studied the effect of thyroparathyroidectomy and parathormone administration on the gastric motility in dogs (Tsai, 1930).

In the autumn of 1930, with funding from the Rockefeller Foundation, Tsai went to the Psychophysiology Laboratory in London. Under the supervision of Prof. C. L. Evans, he studied the changing situation of glycogen in cat livers under different conditions (Evans et al., 1931a, 1931b), as well as the action of adrenaline on glycogen distribution in the cat (Evans et al., 1931c). Afterwards, Tsai studied the action of narcotics on the conduction of nerve impulses from a single end–organ (Tsai, 1931) at E. D. Adrian’s physiological laboratory, Cambridge University, the diagram of neuropotential of which cited by Starling’s Principles of Human Physiology and Harris’ Experimental Physiology.

In the winter of 1931, Tsai briefly visited Goethe University Frankfurt and the other famous physiological laboratories in Germany. From the spring of 1932 to the winter of 1936, Tsai was employed as a researcher in the field of physiology at the Henry Lester Institute of Medical Research, a British-owned institution in Shanghai. During this period, he published 11 research articles in the Chinese Journal of Physiology from 1933 to 1937. Tsai mainly studied the liver of animals. He explored the question of total carbohydrate content of the liver tissue in the fasting rabbit (Tsai, 1933a), and, with the cooperation of assistant Chien Lung Yi (易见龙), taking the liver of the decapitated cat (Tsai, 1933b; Tsai and Yi, 1934a, 1934b, 1934c) and normal intact cat (Tsai and Yi, 1936a, b) as the research object, explored the role of the liver in carbohydrate metabolism on the premise of maintaining the normal blood glucose concentration, which were published in Volumes 8–10 of *The Chinese Journal of Physiology*. Then he explored the question of the validity of using aqueous extracts for estimating glycogen and total carbohydrate of the liver (Tsai, 1934). Based on the study of liver glycogen above, Tasi attempted to study the combined glycogen of liver and muscle (Tsai, 1937a; Tsai, 1937b). Finally, Tsai tried to improve angiostomy (Tsai, 1935). Finally, Tsai tried to improve angiostomy (Tsai, 1935).

In the autumn of 1936, Tsai waived his contract with the Henry Lester Institute of Medical Research one year ahead of schedule, and became a professor of physiology in the newly established Medical College of National Central University in Nanjing. Six months later, the July 7th incident happened, and Tsai moved to Chengdu together with the Medical Department of Central University. His contributions in Chengdu from 1937 to 1945 can be summarized as below: Firstly, Tsai manufactured experimental apparatus. He set up mechanical rooms in Medical Colleague, built equipment, such as soil lathes and drilling machines, which are a pivotal issue to physiological teaching and scientific research and purified pharmaceutical reagents for teaching and research. Secondly, he established academy. In the autumn of 1938, the Chengdu Branch of Chinese Association for Physiological Sciences was founded by Tsai and O. L. Kilborn, a physiology professor at West China Medical University. In the same year, Tsai organized the establishment of the institute of physiology to train trainees, among whom were secretly sent from the Yan’an Military Medical School, such as aeronautical physiologist Huishih Fang (方怀石). Thirdly, Tsai founded a journal to promote academic exchanges of the physiological science field among southwest China, domestic and overseas. Proceedings of Chinese Physiological Chengtu Branch started the publication work in June 1941, Tsai served as editor-in-chief, from then till June 1945, with 13 issues published in total. And this journal was the only physiological academic publication in the rear of Anti-Japanese War. Finally, Tsai conducted blood research. Tsai, as the first author, participated in the academic writing of 11 papers, and guided students to publish more than 20 papers. Three themes were included in these papers: 1. Study on causes of increased erythrocytic fragility (Tsai et al., 1940, Tsai et al., 1941, 1942, Puh et al., 1945). 2. Study on haemolytic and antihaemolytic (Lee and Tsai, 1942a, Lee and Tsai, 1942b, Lee and Tsai, 1943). 3. Study on Chinese hematology standards (Wu and Tsai, 1940) and the standardization of Chinese people’s sensory measurements (Wu and Tsai, 1943). In 1948, Gregerson, President of the American Bureau for Medical Aid to China, led professionals to China to hold a research class to teaching blood related technology. Having had the laboratory led by Tsai as a base, they organized the members of the research class to determine the blood coefficients of dozens of young Chinese.

In 1943, accompanied by another six professors which included Xiaotong Fei (费孝通), Tsai went to America with the identity of Exchange Professor for one year. Tsai introduced nutritional problems of China, with respect to the economic situation, agriculture development, healthy habits and education problems, towards American people (Liu et al., 1946). Also, he studied vasoconstrictor substances with two scholars in the Physiology Department, Medicine Colleague, Columbia University (Tsai et al., 1944), which promote the finding of 5-hydroxytryptamine (Zhang, 2013).

Tsai’s earlier stage of academic work can be recognized from 1924 to 1948, whereby his research topic was foundation physiology. After 1948, his research turned to aviation physiology, navigation physiology with the need for national defense construction.

**Figure 4:**
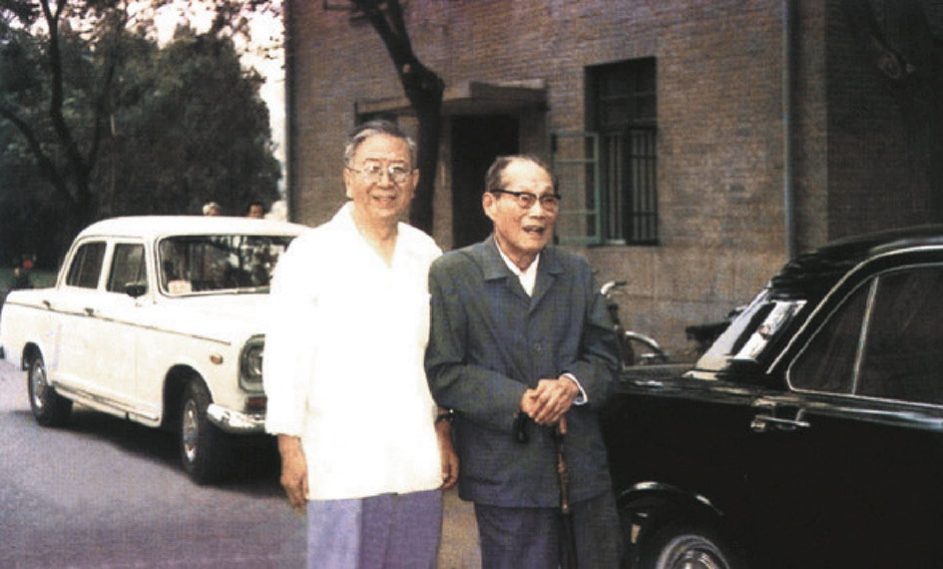
In 1986, Tsai (right) and Zhijun Wang, a physiologist, who was elected as academician in 1980 (Photo credit: Academician Library of Chinese Academy of Sciences https://yswk.las.ac.cn/)

In 1948, Academia Sinica appraised and elected a total of 81 academicians, Tsai was one of 25 academicians in the biology group (Fig. 4). And appointed as the Dean of the medical superintendent of Nanjing University in 1949, principal of The Fifth Military Medical University in 1952, as a leader, he built the concrete human hypobaric chamber, which promoted the development of altitude hypoxia experiments (Zhang, 1997). From 1952 to 1954, Tsai guided young and middle age science and technology personnel and graduates to conduct psychological research including the topic of sleep and pilots’ nutritional situation, which composed the first *Summary of preliminary report on aviation physiology research*.

**Figure 5:**
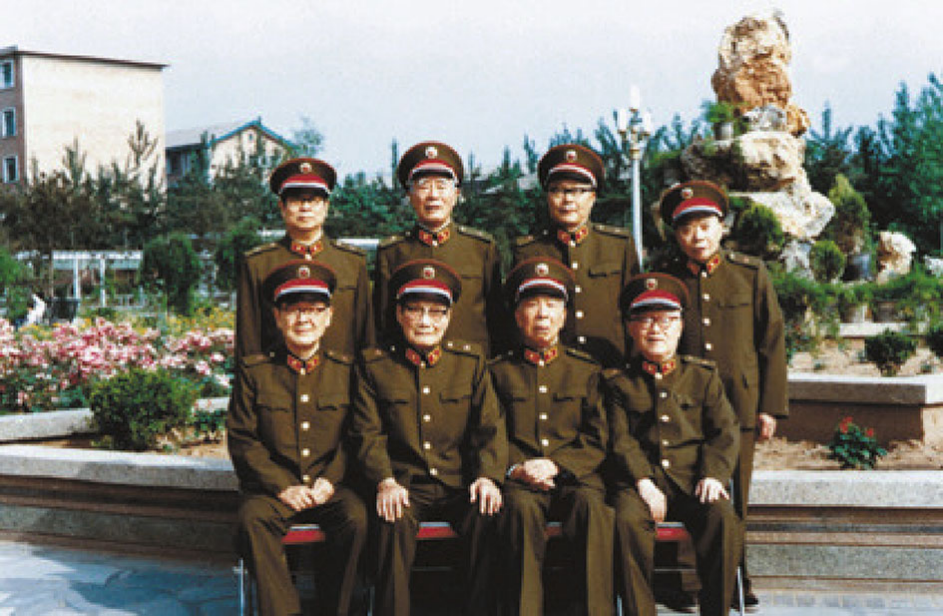
In 1985, Tsai (front row, second from left) and colleagues from the Academy of Military Medical Sciences of the Chinese People's Liberation Army (PLA) (Photo credit: Academician Library of Chinese Academy of Sciences https://yswk.las.ac.cn/)

**Figure 6:**
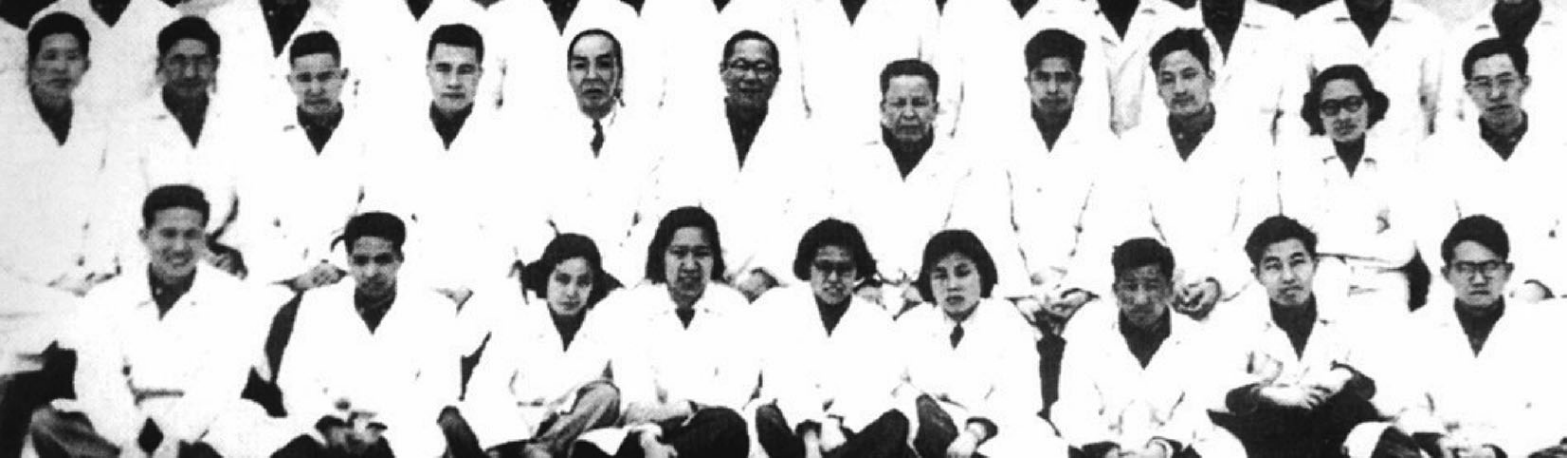
Group photo of teachers and students taken in the early day of the establishment of the Academy of Military Medical Science of PLA (Tsai is in the second row, sixth from the right) (Photo credit: Academician Library of Chinese Academy of Sciences https://yswk.las.ac.cn/)

**Figure 7:**
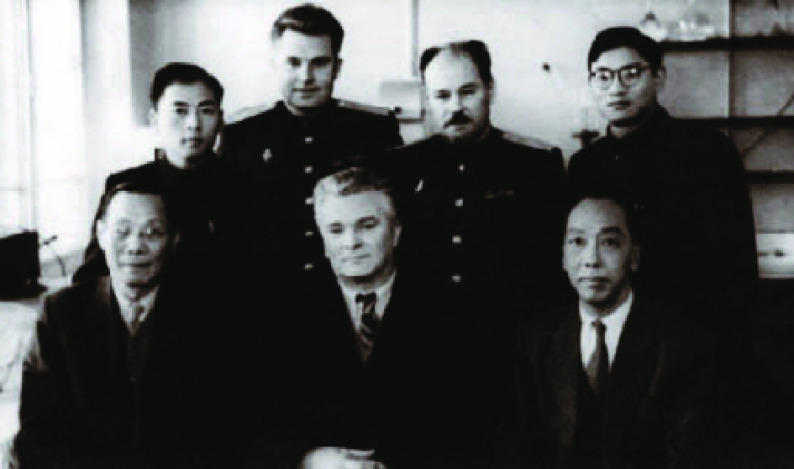
In 1956, Tsai (front row, first from the left) took a group photo with Soviet physiologists in the physiology department of the Kirov Academy of Military Medical Sciences in Leningrad, Soviet Union (Photo credit: Academician Library of Chinese Academy of Sciences https://yswk.las.ac.cn/)

**Figure 8:**
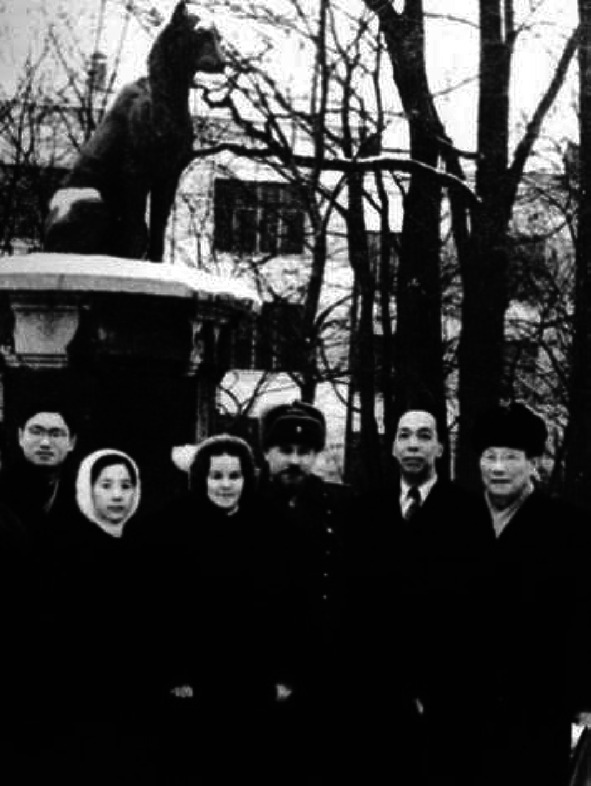
In 1956, Tsai (first from the right) visited the Soviet Union and took a photo in front of Leningrad Institute of Experimental Medicine (Photo credit:Academician Library of Chinese Academy of Sciences https://yswk.las.ac.cn/)

Between 1954 and 1968, Tsai, on basis of the nomination from Central Military Commission, became vice-president of the Academy of Military Medical Sciences of PLA (Figs. 5 and 6) and director of Military Performance and Physiology Institute which was renamed the Aerospace Medicine Institute in 1964. Between 1957 and 1966, Tsai leaded people to build numerous large scale pieces of equipment and formulated safeguard regulations and device requests suitable for the Chinese Military. At the same time, Tsai took the role of vice-president of Academy of Military Medical Sciences of PLA, and leaded research to conduct scientific research related with aviation physiology. Then they wrote 12 papers for the *Chinese People*’*s Liberation Army Academy of Military Medical Sciences Proceedings*, for example, with the theme of tentative modelling and identification of flight fatigue, the human body’s tolerance of high and low temperature. Tsai also took part in the International Physiological Science, Brussels Conference in 1956. From August, 1956 to February, 1957, as the deputy head of the Chinese Military Medical Delegation, Tsai conducted an integrated survey in the Soviet Union for around six months (Figs. 7 and 8), then he participated in international conferences in Czechoslovakia and Poland. Tsai also attended the International Space Science Conferences in Warsaw, International Aviation Space Medicine Conference in Ireland, and he also visited a research laboratory in London. In 1966, China made the cosmomedicine plan drafted by Tsai, and this led to the establishment of the Cosmomedicine Medical Engineering Institute, which contributed to the development of China’s cosmomedicine career.

**Figure 9:**
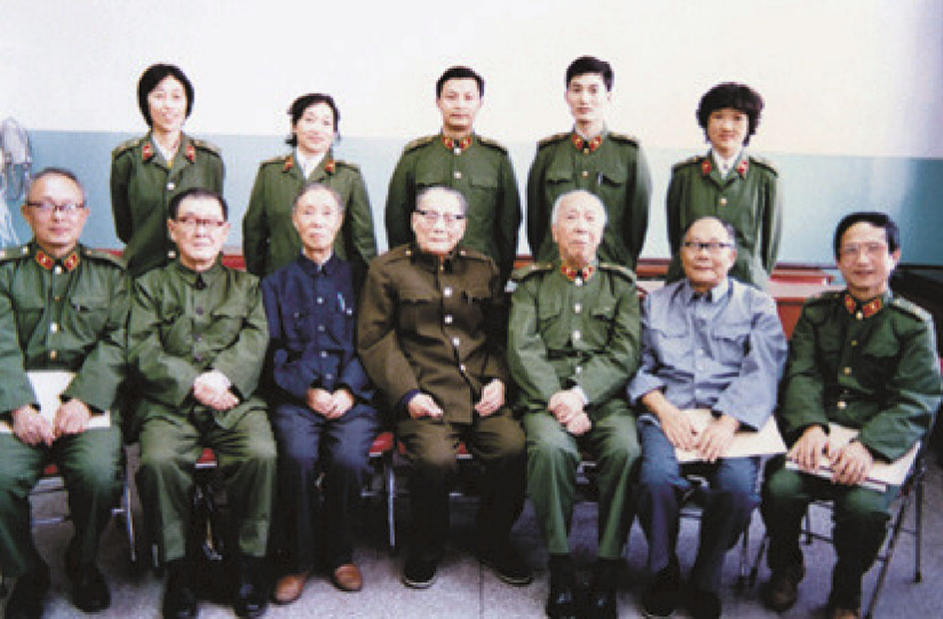
In September, 1986, Tsai (front row, in the middle), as the judge of doctoral thesis defense, took a photo with the jury and the Ph.D graduates after the defense meeting (Photo credit: Academician Library of Chinese Academy of Sciences https://yswk.las.ac.cn/)

From 1966 to 1976, Tsai lost his medical career, but still insisted on writing *Fundamentals of Aviation and Space Medicine*, which was published in 1979. In 1978, Tsai reclaimed his title as vice-president at the Academy of Military Medical Science of PLA, where he guided research in the neurobiology laboratory and trained postgraduates (Fig. 9). In 1981, Tsai retired from the Chinese Association for Physiological Sciences with the position of president of a council. On July 29th, 1990, Tsai passed away.
